# S100/RAGE-Mediated Inflammation and Modified Cholesterol Lipoproteins as Mediators of Osteoblastic Differentiation of Vascular Smooth Muscle Cells

**DOI:** 10.3389/fcvm.2018.00163

**Published:** 2018-11-08

**Authors:** Bijoy Chellan, Nadia R. Sutton, Marion A. Hofmann Bowman

**Affiliations:** ^1^Department of Medicine, University of Illinois, Chicago, IL, United States; ^2^Department of Medicine, University of Michigan, Ann Arbor, MI, United States

**Keywords:** smooth muscle, S100, RAGE (receptor for AGE), calcification, modified LDL

## Abstract

Arterial calcification is a feature of atherosclerosis and shares many risk factors including diabetes, dyslipidemia, chronic kidney disease, hypertension, and age. Although there is overlap in risk factors, anti-atherosclerotic therapies, including statins, fail to reduce arterial, and aortic valve calcifications. This suggests that low density lipoprotein (LDL) may not be the main driver for aortic valve disease and arterial calcification. This review focuses on modified LDLs and their role in mediating foam cell formation in smooth muscle cells (SMCs), with special emphasis on enzyme modified non-oxidized LDL (ELDL). *In vivo*, ELDL represents one of the many forms of modified LDLs present in the atherosclerotic vessel. Phenotypic changes of macrophages and SMCs brought about by the uptake of modified LDLs overlap significantly in an atherosclerotic milieu, making it practically impossible to differentiate between the effects from oxidized LDL, ELDL, and other LDL modification. By studying *in vitro*-generated modifications of LDL, we were able to demonstrate marked differences in the transcriptome of human coronary artery SMCs (HCASMCs) upon uptake of ELDL, OxLDL, and native LDL, indicating that specific modifications of LDL in atherosclerotic plaques may determine the biology and functional consequences in vasculature. Enzyme-modified non-oxidized LDL (ELDL) induces calcification of SMCs and this is associated with reduced mRNA levels for genes protective for calcification (ENPP1, MGP) and upregulation of osteoblastic genes. A second focus of this review is on the synergy between hyperlipidemia and accelerated calcification *In vivo* in a mouse models with transgenic expression of human S100A12. We summarize mechanisms of S100A12/RAGE mediated vascular inflammation promoting vascular and valve calcification *in vivo*.

## Intimal and medial calcification: two distinct pathologic processes?

Two main types of vascular calcification have been reported in adults: intimal calcification and medial calcification (also known as Mönckeberg sclerosis). Intimal calcification occurs in the setting of lipid accumulation, foam cell formation, and macrophage infiltration, and is often present in advanced atherosclerotic plaques. In contrast, medial calcification occurs without intimal plaque formation and localizes to elastin fibers and smooth muscle cells (SMCs); foam cells and infiltrating macrophages are not essential to formation of medial calcification. Medial calcification can and does occur independent of intimal plaque formation in some cases, but the processes are not always separate and medial calcification can be found in patients that also have intimal calcification of plaques. For example, calcification of coronary arteries occurs mainly in the intima and is strongly associated with increased risk of future myocardial infarction. Hence, coronary artery calcification (CAC) on computed tomography is associated with cardiovascular morbidity and mortality (1). Screening for CAC can be considered for asymptomatic individuals if uncertainty remains after conventional risk assessment, since CAC adds incremental value for cardiovascular risk prediction (2).

In contrast, thoracic aortic calcification (TAC) preferentially involves the media and provides limited incremental value for cardiovascular risk, and current data do not support screening for TAC (3). TAC is more associated with non-cardiovascular morbidity and non-cardiovascular mortality, and may be general marker of biological aging as recently reported by Thomas et al. (4). In this study, TAC was analyzed in 6,765 participants in the Multi-Ethnic Study of Atherosclerosis (MESA, median age 62), a prospective cohort study of subclinical atherosclerosis, in which participants underwent computed tomography at baseline and followed longitudinal for median 12.2 years. TAC in the highest tertile (compared to no TAC) was associated with a higher risk of non-cardiovascular mortality (HR 1.56 [1.23–1.97]), as well as an increased risk for hip fracture (HR 2.14 [1.03–4.46]), chronic obstructive lung disease (HR 2.06 [1.29–3.29]), and pneumonia (HR 1.79 [1.3–2.45]), with magnitudes of associations that were larger than those for coronary artery calcium.

Another example of medial calcification is the “tram-track sign” that can be seen on routine mammography X ray for breast cancer screening in 60–70% of women>70 years of age or with chronic kidney disease. This represents calcification along the circumference of the internal mammary artery. Calcification of the internal mammary artery is associated with cardiovascular disease with the highest hazard ratio for heart failure (HR 1.52 [1.18–1.98]) and to a lesser degree with coronary artery disease (HR 1.29 [1.01–2.05]) (5, 6). Similarly, a study by Margolies et al. provided a detailed assessment of the association of calcification in the breast arteries and coronary arteries by studying 292 women referred for screening mammogram and clinically indicated chest computed tomogram. After adjusting for age and traditional risk factors, only the presence of moderate to severe breast artery calcification (HR 3.2; [1.8–5.9]), age (HR 2.0 [1.5–2.6]), and hypertension (2.2 [1.3–3.8]) remained significant predictors for the presence of CAC (7). These and other epidemiological findings underscore important clinical differences between atherosclerotic intimal calcification and medial calcification, but at the same time provide evidence for co-existence and shared risk factors.

**Figure 1:**
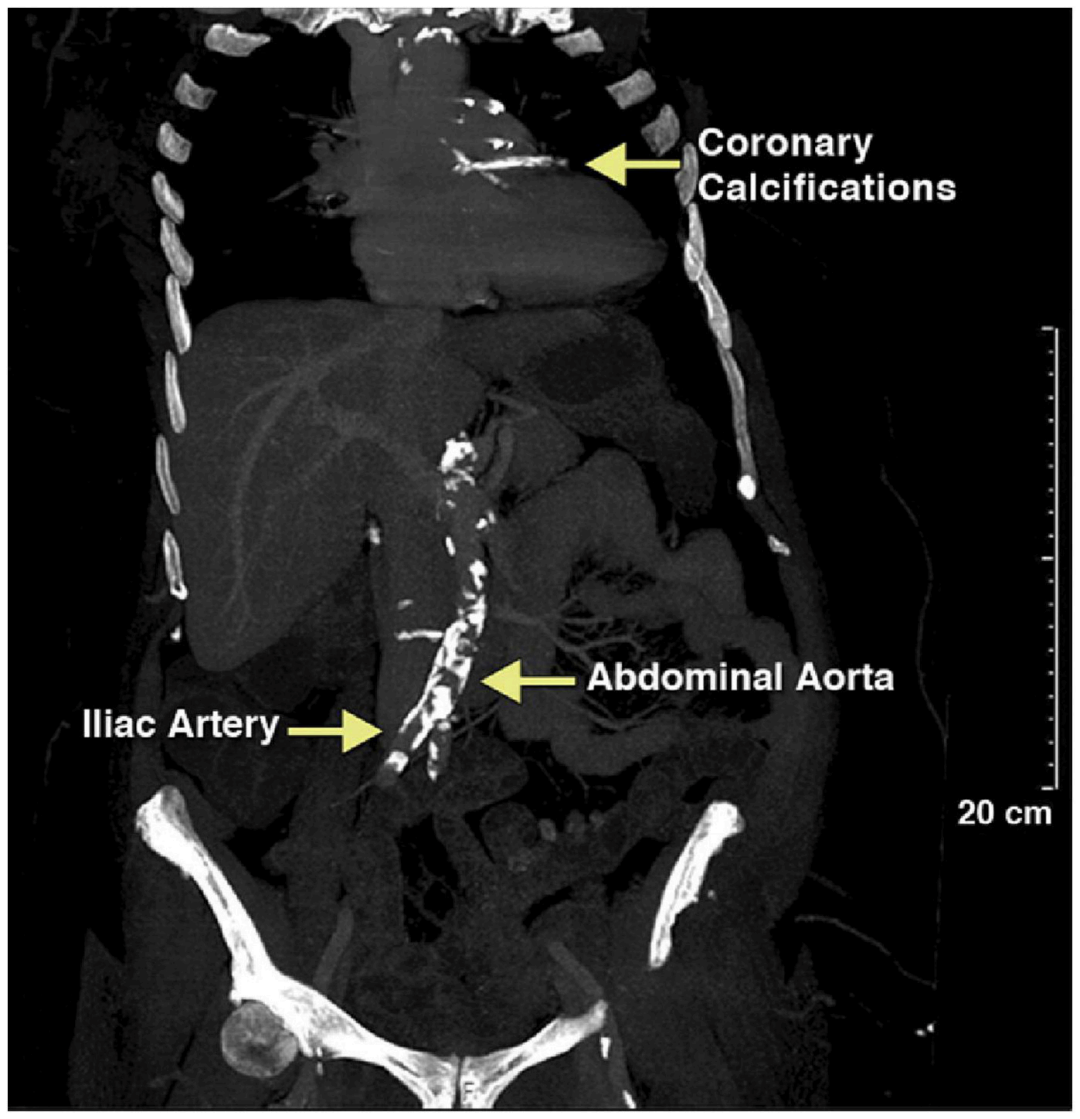
Calcified vessels in a female mummy, estimated age 40–45 years old, a princess living in the Seventeenth Dynasty (1580–1550 BCE) of the Second Intermediate Period. Extensive calcification of the aortic and coronary vessels was detected using CT scanning. Reproduced with permission from Elsevier from the Horus Study published in JACC Cardiovascular Imaging (8).

Vascular calcification in adults becomes more prevalent with age, diabetes mellitus, dyslipidemia, renal disease, bone-mineral disorders, and hypertension and is readily detectable by multiple imaging modalities. Computed tomography of mummified ancient Egyptians have documented a high prevalence of calcification of the coronary, carotid, iliac, femoral, and peripheral leg arteries and extending to the aorta, indicating that calcification is not a modern disease (Figure 1). In the last four decades we have made substantial progress to understand the mechanisms contributing to this ancient disease. Particular, the discovery that alkaline phosphatase (ALPL), an enzyme critically important for calcification of cartilage and bones, is present in matrix vesicles at the site of atherosclerotic and medial vascular mineralization has provided intriguing evidence that actively regulated osteogenic processes are participating in vascular mineralization (9). These earlier studies were followed by landmark discovery by Boström et al. who in 1993 demonstrated that bone-morphogenetic protein 2 (BMP2) is expressed in calcified human atherosclerotic plaques (10), and followed by later studies showing that BMPs direct osteogenic programming of vascular cells. Briefly, BMPs are secreted polypeptides and belong to the subgroup of transforming growth factor β superfamily. BMPs elicit their effects through activation of receptor complexes composed of type I and type II Ser/Thr kinase receptors to activate smad-signaling pathways as well as non-smad signaling pathways, ultimately switching on an osteochondrogenic gene program [review by Bostrom et al. (11)]. A causal relationship between BMP activity and vascular calcification has been established (12–14). Thus, in a span of 2 decades a picture emerged in which the mineralizing pathobiology of vascular calcification is mediated by the same osteochondrogenic factors that are responsible for bone morphogenesis during skeletal development and fracture repair. Our knowledge about factors that play a role in the phenotypic switch of vascular cells to gain features of “osteoblast-like cells” has increased substantially over the years, and there is a growing list of physiologic modulators and environmental factors that contribute to intimal calcification in atherosclerosis and to calcification of the tunica media. These physiological and environmental factors accumulate in patients with chronic kidney disease, and chronic kidney disease has been associated with profound acceleration of vascular calcification. For more details on the impact of uremic toxins on vascular SMC proliferation, migration, cell death, calcification, and senescence we refer to a timely review paper on this topic (15). Although there is experimental evidence that cells ultimately linked to vascular calcification are from multiple sources including endothelial cells, monocytes, macrophages, dendritic cells, progenitor cells, fibroblasts, and SMCs, there is growing evidence that the phenotypic switch from contractile to synthetic SMC plays an important role in vascular calcification. Our understanding of the plasticity of SMC has grown substantially over the last decade and for more details we refer to recent review papers (16–18). This review here focuses on the physiologic modulator S100A12 and its synergistic effects with modified low density lipoproteins (LDLs) in promoting osteochondrial differentiation of SMC.

## Transgenic overexpression of S100A12 as a model for vascular calcification and translation to human disease

Studies on the molecular mechanisms of vascular calcification have relied on cell-based and animal model systems. Common rodent models for atherosclerotic disease are hyperlipidemic mice, either deficient in apolipoprotein E or low-density lipoprotein receptor fed a high-fat diet [reviewed in (19, 20)], while mouse models for medial calcification commonly utilize mice with normal lipid levels subjected to uremia [5/6th nephrectomy, or ureter ligation model, or other renal injury, reviewed in (21, 22)]. Increasingly, transgenic mice with altered expression of factors such as matrix Gla protein [MGP (23)], Klotho (24, 25), BMP4 (26), Ectonucleotide Pyrophosphatase/Phosphodiesterase 1 (ENPP1) (27), and others have been generated, vastly improving our understanding of mechanisms leading to SMC calcification.

Our laboratory has developed a novel model of accelerated vascular calcification by transgenic expression of human S100A12 targeted to smooth muscle by using the SM-22 promoter. These animals were created to probe whether S100A12 causes atherosclerosis, since many human cohort studies showed an association of S100A12 with atherosclerosis as outlined below. S100A12 is endogenously expressed in myeloid cells and other cells and has pleotropic effects, including antimicrobial properties and thereby plays a key role in host defense (28, 29). Similar to other antimicrobial proteins, S100A12 has been shown to modulate immune responses. S100A12 activates RAGE, the receptor for advanced glycation end products (30, 31), and toll-like receptor 4 (32). Once activated, RAGE initiates a cascade of downstream signals including activation of transcription factor NFκB, activation of ERK1/2, MAPK, IL-1β, IL-6 (30, 33).

A potential role of S100A12 in mediating plaque instability or acute myocardial infarction is supported by epidemiological data. For example, upregulation of S100A12 in macrophages and in SMCs was found in ruptured atherosclerotic plaques causing sudden cardiac death compared with stable coronary artery plaques (34). Serum levels of S100A12 have been associated with atherosclerosis in many cohort studies (35), and the Rotterdam study was the first longitudinal study to suggest a causal role of S100A12 for coronary artery disease (36). In this study with 10 year follow up, baseline S100A12 serum concentrations had predictive value for development of coronary artery disease, acute myocardial infarction, and cardiovascular mortality in this population-based cohort study of 839 participants without coronary heart disease at baseline. S100A12 remained significantly associated with coronary artery disease when adjusted for age and sex. Participants in the highest tertile of S100A12 levels had a 2.6-fold higher risk of developing coronary artery disease compared with participants in the lowest tertile. After further adjustments for other inflammatory markers (hs-CRP, CD40, IL-8, IL-18, and other inflammatory markers) and for traditional risk factors (diabetes, hypertension, chronic kidney disease, smoking, body mass index, hyperlipidemia, white blood cell count), S100A12 remained associated with acute myocardial infarction and cardiovascular mortality, suggesting that S100A12 represents a distinct inflammatory pathway related to athero-thrombosis. Further, S100A12 mRNA in peripheral blood cells was one of the most significantly reduced genes (among 142 other genes that were differentially expressed from baseline) in 63 participants with coronary artery disease or ≥2 CAD risk factors after 52 weeks of a rigorous cardiovascular disease risk reduction program with comprehensive life style changes (37), demonstrating that S100A12 expression in leucocytes is a modifiable risk factor. Moreover, in patients with end stage renal disease on hemodialysis, circulating S100A12 levels are associated with cardiovascular mortality (38, 39), progression of abdominal aortic calcification (40) and with progression of CAC (41).

**Figure 2:**
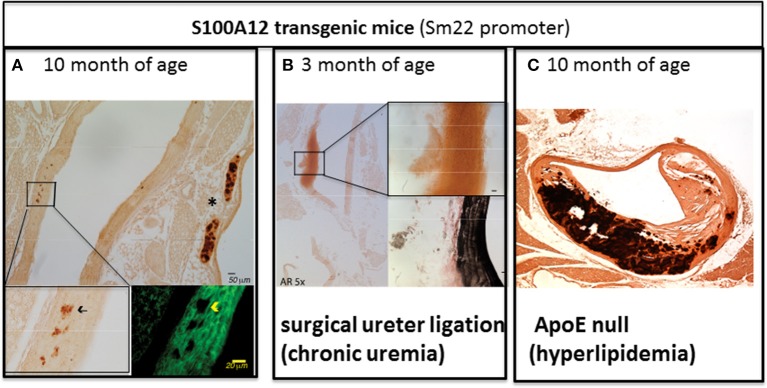
The amount of aortic vascular calcification visualized by Alizarin Red Staining in transgenic mice with S100A12 expression targeted to smooth muscle is modulated by environmental factors. **(A)** Scant medial calcification in the aorta of 10-month old S100A12/C57Bl6J mice with insert on the lower left for Alizarin Red stain and insert on the lower right with immunofluorescent microscopy staining alpha smooth muscle actin in green. *marks calcification of cartilage of the trachea. Not pictured are wild type C57Bl6 littermate controls, which had no medial calcification; Reproduced with permission from Lippincott Williams and Wilkins (42). **(B)** Large medial calcification in the aorta of S100A12/C57Bl6J mice with chronic uremia induced by ureter ligation and with insert on the upper right for Alizarin Red stain and insert lower right with Verhoeff van Gieson staining elastic fibers in black. Not pictured are wild type C57BL6J littermate controls with chronic uremia which had no medical calcification; Reproduced with permission from Karger Publishers (43). **(C)** Advanced atherosclerosis and calcification of atherosclerotic plaques in S100A12 transgenic/ApoE null mice. Not pictured are WT/ApoE littermate controls which had reduced atherosclerosis and calcification; Reproduced with permission from Lippincott Williams and Wilkins (44).

While normal SMCs do not express S100A12, SMCs in atherosclerotic plaque and particular in ruptured plaques associated with sudden cardiac death express S100A12 (34). To study the role of S100A12 in SMCs, we generated transgenic mice with expression of human S100A12 under control of smooth muscle promotor SM22α, exploiting the fact that mice lack the gene for S100A12. In C57BL6 mice with normal cholesterol, S100A12 transgenic mice develop calcification of the medial smooth muscle layer in the absence of atherosclerotic plaques with aging [Figure 2A (42)] or at younger age when subjected to chronic uremia [Figure 2B (43)]. One mechanism by which S100A12 mediates vascular medial calcification involves enhanced oxidative stress. We found upregulation of Nox-1 mRNA and protein in the aorta of S100A12 transgenic mice, and importantly, silencing of Nox-1 in isolated SMCs from S100A12 transgenic mice resulted in attenuation of *in vitro* calcification together with reduction of BMP-2 mRNA levels. This suggests that NADPH-derived reactive oxygen species mediate the pro-calcific effects of S100A12 in SMCs (43). Enhanced oxidative stress is well-established as an important cause of vascular calcification and this is mediated, at least in part, by activation of oxidative stress sensible transcription factor Runx-2 (45). Runx-2 is a master regulator of bone development. Recent work demonstrated that Runx-2 expression in smooth muscle is required for medial calcification in mice, since targeted deletion of Runx-2 in smooth muscle prevented the severe arterial calcification induced by high dose vitamin D (46). Translocation of NFκB, another master regulator of gene transcription including pro-inflammatory and osteoblastic genes, is also regulated by the S100/RAGE/oxidative stress axis (47). RAGE signaling has been implicated to augment vascular calcification (48–50). Although RAGE deficiency attenuates atherosclerosis in ApoE null mice (33, 51), effects on vascular calcification have not been reported in RAGE/ApoE null mice.

As outlined above, S100A12 is sufficient to promote medial arterial calcification in a non-atherosclerotic manner. However, when S100A12 transgenic mice were crossed with atherosclerosis prone ApoE null mouse, we noted an increase in atherosclerotic plaque size characterized by a very large increase in calcified intimal plaque and in medial calcification in S100A12/ApoE null compared to their wild type/ApoE null littermate mice (Figure 2C) (44). This accelerated vascular calcification in the S100A12/ApoE null mice demonstrates the synergistic effects of S100A12 and hyperlipidemia in promoting calcification. The vascular pathology observed in S100A12/ApoE null mice consisting of intimal calcification, large necrotic core, and breakdown of elastic fibers models features that are seen in human coronary artery disease.

In the S100A12/ApoE null mouse model with accelerated vascular calcification we identified a phenotypic switch from contractile SMC to osteoblast-like cells. Even before calcification could be visualized by Alizarin red staining, we found increased levels of osteoblastic gene expression including BMP-2, BGLP, Dentin matrix acidic phosphoprotein 1 (DMP-1), and Runx-2 in the aorta of S100A12/ApoE null mice supporting a role for S100A12 to induce a phenotypic switch that drives cells to an osteoblast-like cell *in vivo*. In cultured SMCs isolated from the aorta of young S100A12 transgenic mice, S100A12 transgene expression alone was not sufficient to induce an osteoblast-like cell transformation and/or spontaneous calcification of SMCs cultured in regular DMEM medium, but gained osteoblast-like features when hyperlipidemic serum from ApoE null mice was added to the cell culture medium. This strongly suggests that S100A12 primes SMCs to switch to osteoblast-like cells in the presence of lipoprotein cholesterol (44).

In summary, transgenically-expressed S100A12 mediates vascular calcification and pathological remodeling of the blood vessel, but this process is dependent on environmental cues with hyperlipidemia being an important modulator of inflammation-driven vascular calcification in this model. This is illustrated by comparing the amount of arterial calcification between Figures 2A,C, both images depict the vasculature of 10-month old S100A12 transgenic mice maintained on a normal rodent chow diet, with Figure 2A depicting a ApoE^+/+^ mice and Figure B depicting a hyperlipidemic ApoE^−/−^ mice. Taken together, these findings suggest that cholesterol lipoproteins have profound effects on SMCs by altering their function with gain in osteoblast-like phenotype. We speculate that the pro-inflammatory milieu mediated by activation of the S100/RAGE axis might modify lipoproteins and render them more atherogenic. Our lab recently reported on mechanisms of transition of SMCs into foam cells with osteoblast-like differentiation in response to enzyme-modified LDLs as outlined below.

## Foam cell formation in SMCs

The uptake of cholesterol lipoproteins by cells leads to morphological changes visible by light microscopy, and this was termed “foaming,” or “foam cell formation.” Foam cells are a pathological hallmark of atherosclerosis. Since foam cells express several macrophage markers, it has long been thought that foam cells arise primarily from monocyte-derived macrophages. Rong et al. demonstrated in 2003 that SMCs in response to cholesterol loading downregulate expression of typical SMC markers such as smooth muscle actin and upregulate markers commonly seen in macrophages (CD68) (52). Recent discoveries suggest that SMCs are an important source of foam cells in atherosclerosis. Notably, human arteries compared to murine arteries are enriched with SMCs. For example, the medial layer of the human aorta contains approximately 50–60 lamellar units of SMCs sandwiched between elastic fibers (53) compared to approximately only eight laminar units in mice. Recent studies demonstrate that as many as 50% of foam cells in human and murine lesions are derived from SMCs (54, 55), confirming histopathological studies performed three decades earlier that already suspected SMCs as a significant source for intimal foam cells (56).

These findings have sparked our interest in understanding the mechanisms by which lipoproteins are taken up by SMCs and mediate phenotypic switches toward the development of atherosclerosis and calcification. There are several lines of evidence suggesting that native LDL is not the primary driver of medial calcification. First, statins have a marginal effect on reducing vascular calcification, as noted in several clinical trials, despite their ability to lower serum LDL (57). Secondly, statins have been studied in four prospective randomized clinical trials for calcific aortic valve disease, and did not resulted in slowing progression of aortic stenosis. Studies in our laboratory have investigated native LDL and different forms of modified LDL [oxidized LDL, acetylated LDL, and enzyme-modified non-oxidized LDL (ELDL)] and their ability to foam SMCs and induce phenotypic changes including gain of osteoblast-like functions as outlined below. Modified LDLs exists in multiple forms, characterized by different degree of changes of the lipoproteins, cholesterol and fatty acids. Even when identical conditions are used to oxidize the LDL *ex vivo*, the products could differ significantly, depending on the fatty acid composition and antioxidant status of the starting LDL preparation. The diversity of oxidized LDL particles is reviewed by Levitan et al. (58) and for enzyme modified LDL by Torzewski (59).

**Figure 3:**
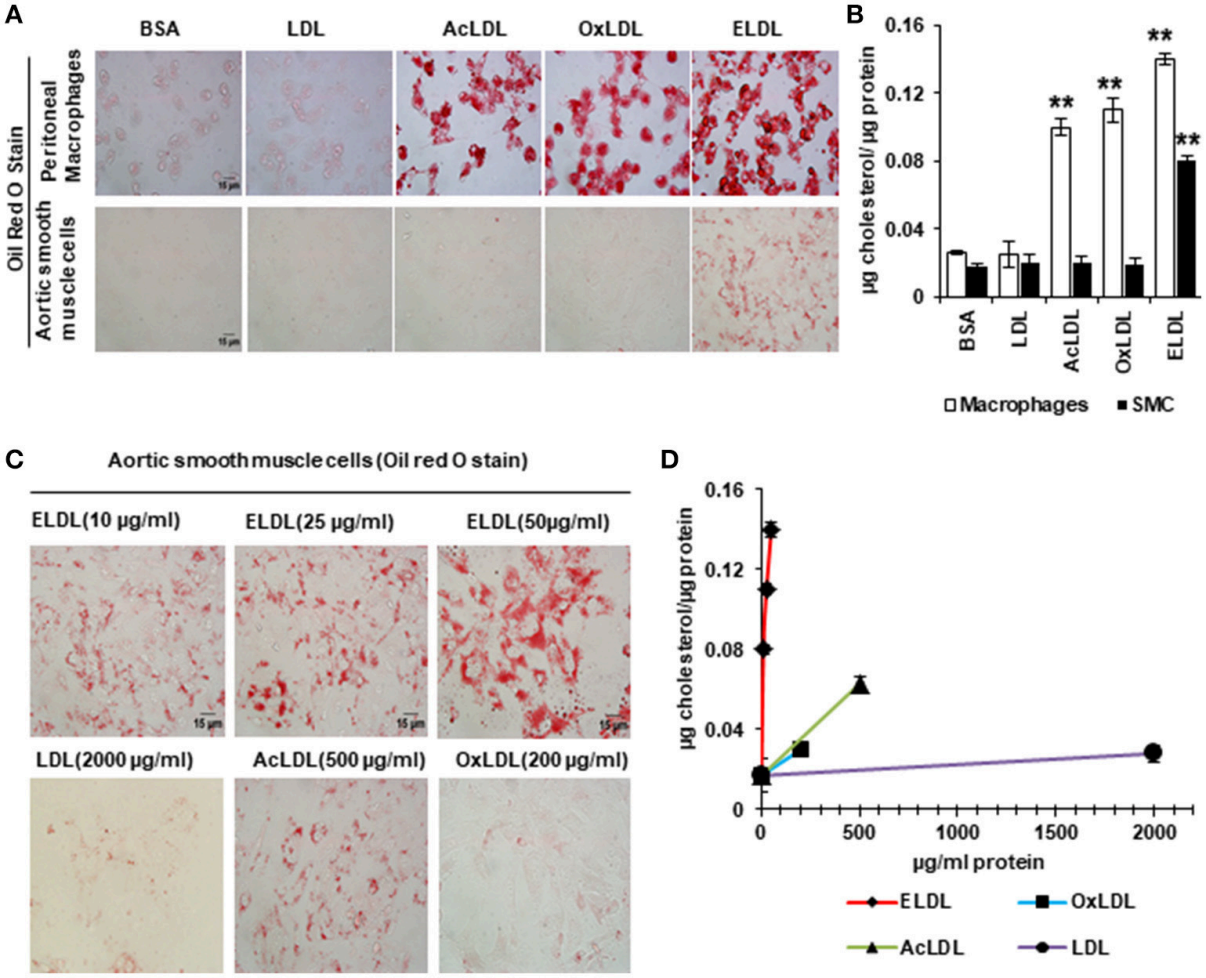
Enzyme modified LDL (ELDL), but not acetylated (acLDL) or oxidized LDL (oxLDL) induces foam cell formation in murine SMCs, in contrast to macrophages which readily take up acLDL, oxLDL and ELDL. In, **(A)** Oil Red O staining for peritoneal macrophages (upper) and for aortic SMCs (lower) stimulated with 10 μg/ml modified LDL as indicated. **(B)** Quantification of intracellular cholesterol. **(C)** Oil Red O staining of aortic SMC stimulated with different concentration of LDLs as indicated and quantified in **(D)**. Reproduced with permission from Lippincott Williams and Wilkins (61). **p < 0.01.

In spite of the emerging evidence that vascular SMCs contribute significantly to the pool of foam cells in atherosclerotic lesions, little is known about the mechanisms of foam cell formation in SMCs. Scavenger receptor-dependent endocytosis is the principle route for LDL uptake by macrophages with CD36, SRA1, and LOX-1 acting as the major scavenger receptors involved in this process (60). Although SMCs also express scavenger receptors and acquire macrophage-like phenotypes upon lipid loading (gain of expression of CD68 and Mac2 and loss of expression of alpha actin and alpha tropomyosin) (52), our laboratory recently demonstrated that the mechanism of cholesterol loading differs between SMCs and macrophages. *In vitro*, SMCs preferentially uptake ELDL, and uptake of oxidized, acetylated or native LDL occurs only minimally. This is in contrast to macrophages, which readily take up oxidized LDL, acetylated LDL, ELDL, and native LDL (Figure 3). We demonstrated that ELDL uptake by SMC is dependent on macropinocytosis, rather than on scavenger receptors (61, 62). Macropinocytosis is the process whereby the cell membrane folds to form vacuoles and thereby incorporates bulk cargo, such as dissolved molecules, and large volumes of extracellular fluid. Regulators of macropinocytosis are complex, and calcium is required for folding of the cell membrane leading to macropinocytosis (63, 64). In our studies, the calcium channel blocker lacidipine abolished ELDL uptake in SMCs, and RAGE was not required for the uptake of ELDL into SMCs. However, RAGE modulated signals that participated in the regulation of macropinocytosis. We found that pharmacological inhibition of PI3K, the enzyme responsible for phosphoinositide phosphatidylinositol 3,4,5-trisphosphate (PIP3) formation, abolished ELDL uptake in RAGE-deficient SMCs but was only partially effective in preventing the uptake of ELDL in wild type SMCs (61). Although macropinocytosis is the principle mechanism of ELDL uptake in murine aortic SMCs and in human coronary artery SMCs (HCASMCs), we found physiological differences between murine aortic SMC and HCASMCs with regard to ELDL uptake. In murine aortic SMCs, ELDL upregulated LOX-1 mRNA, and protein and this primed murine aortic SMC for the uptake of oxidized LDL (61), however, this was not observed in HCASMCs. ELDL did not upregulate LOX-1 or prime HCASMCs for uptake of oxLDL (unpublished data). This suggests that susceptibility for cholesterol uptake and foam cell formation may depend on the specific vascular origin of cells and/or is species-specific.

ELDL is generated *in vitro* by incubation of native LDL, isolated from human plasma, with trypsin and cholesteryl ester hydrolase. Trypsin is a protease which cleaves the apoB protein, thereby facilitating access for cholesteryl ester hydrolase to the lipid core. A similar process can take place *in vivo*, and cholesteryl ester hydrolase is present in human arterial plaques at concentrations high enough for direct detection by immunostaining (65, 66). Protease enzymes that potentially could act as the modifying protease for LDL *in vivo*, similar to trypsin *in vitro*, could be plasmin, chymases, MMPs, and cathepsins, which are all highly expressed in atherosclerotic plaques. Torzewski et al. have shown that ELDL is present in human atherosclerotic plaques and in calcific aortic valves (67, 68) by using monoclonal antibodies for ELDL that react only with proteolysed apoB. Taken together, these findings suggest an important link by which vascular inflammation promotes the local production of ELDL, which in turn is taken up by SMCs and produces foam cells.

Chronic sustained inflammation in atherosclerotic plaques, at least in part, stems from infiltrating macrophages, which provide an “inflammatory input” to facilitate osteoblastic transformation of vascular cells, including SMC's. This link was described by Tintut et al.; human recombinant TNF-α was sufficient to induce markers of osteoblastic differentiation in cultured bovine aortic SMCs (69). In addition to pro-inflammatory cytokines, oxidative stress, uremia, mitochondrial dysfunction, mechanical stress, and loss of inhibitors all collectively promote an *active* phenotypic switch of SMC to become osteoblast-like cells [reviewed in (18)], *passive* calcification of dead cells, apoptotic bodies, and necrotic debris has also been proposed as an important participant in vascular calcification. Some cells of the fibrous cap and of the lipid rich core stain positive for the apoptosis marker TUNEL and for IL-1β, suggesting that apoptotic cells accumulate in the fibrotic lesions in a “mummified” state (70). Morphological findings in human coronary atherosclerosis include a close spatial relationship of calcification with apoptotic SMCs and macrophages (71). However, which occurs first, calcification or apoptosis, remains unclear, since calcium phosphate deposits, TNFα, S100A12, and many other processes have been shown to induce both, an osteoblastic phenotype as well as apoptosis in vascular smooth muscle. For example, we showed that S100A12 induces expression of Fas, caspase 10, and caspase 3 in human airway SMCs (72) and knockdown of S100A12 in human aortic SMCs harvested from patients with aortic dissections attenuated cell death (73). These findings are consistent with a pro-apoptotic function of S100A12 and in agreement to earlier studies demonstrating that S100A8/9 induces apoptosis and cell death in a variety of cell types (74, 75). Taken together, cell death could either be a result of the initial calcification, or calcification could be a consequence of apoptosis.

## Lipoprotein cholesterol controls transformation of SMCs to osteoblast-like phenotype

*In vivo*, ELDL represents one of the many forms of modified LDLs that form foam cells in macrophages and smooth muscles cells, and phenotypic modulations brought about by individual modified LDLs overlap significantly in an atherosclerotic milieu, making it practically impossible to differentiate between each other. By studying *in vitro*-generated modifications of LDL we were able to demonstrate marked differences in the transcriptome of HCASMCs upon uptake of ELDL, OxLDL, and native LDL, indicating that specific modifications of LDL in the atherosclerotic plaques may determine the biology and functional consequences in vasculature (62). A main finding was that gene expression is distinctly different between HCASMC stimulated with oxidized LDL compared to ELDL or native LDL. For example, of the 1,393 genes differentially expressed in oxidized LDL treated cells, there were overlaps with only 74 genes in ELDL treated cells. Functional annotation cluster and Ingenuity pathway enrichment analysis predicted activation of p38 MAPK, NFκB, and ERK as the top three canonical pathways for ELDL-stimulated HCASMCs, and this was confirmed biochemically. Moreover, bioinformatics network analysis of cardiovascular systems development showed that ELDL-treated HCASMC had activation of several nodes related to SMC differentiation and calcification including canonical pathways of “Role of Osteoblast, Osteoclasts and Chondrocytes in Rheumatoid Arthritis,” “Role of Pattern Recognition Receptors in Recognition of Bacteria and Virus,” and “Atherosclerotic Signaling,” thus providing the rationale to examine whether ELDL promotes mineralization of SMCs.

**Figure 4:**
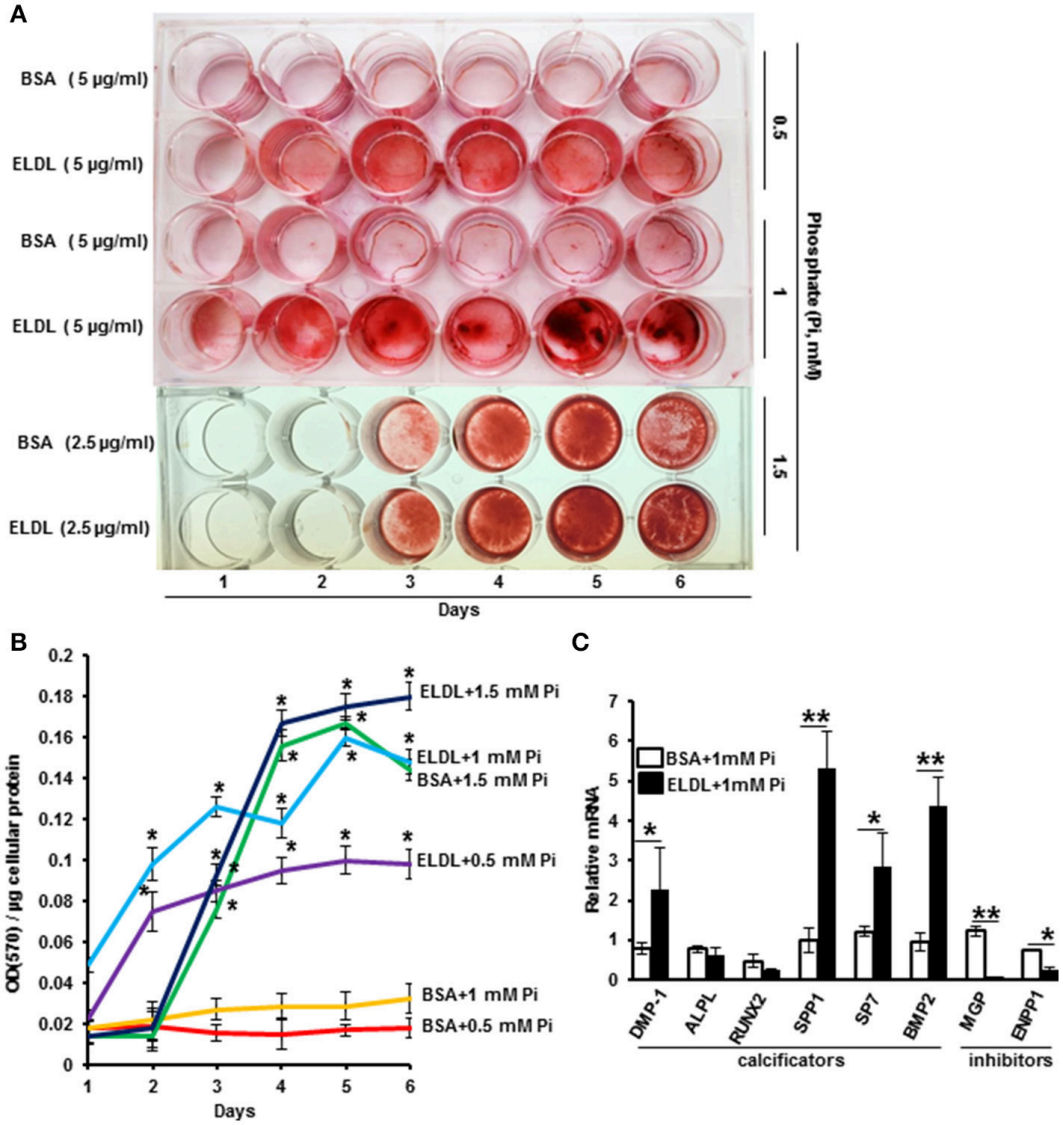
Enzyme modified LDL (ELDL) upregulates phosphate induced calcification in cultured human coronary artery smooth muscle cells. **(A)** HCASMC cultured in phosphate containing medium (as indicated) with either control BSA or ELDL as indicated. Calcium phosphate deposits were stained with Alizarin Red. **(B)** Quantification of Ca^2^ deposits. **(C)** mRNA levels of selected osteoblastic genes in HCASMC incubated for 24 h with either 10 μg/mL ELDL or control BSA with 1 mM inorganic phosphate. Reproduced with permission from Macmillian Publishers Ltd. (62). *p < 0.05; **p < 0.01.

**Figure 5:**
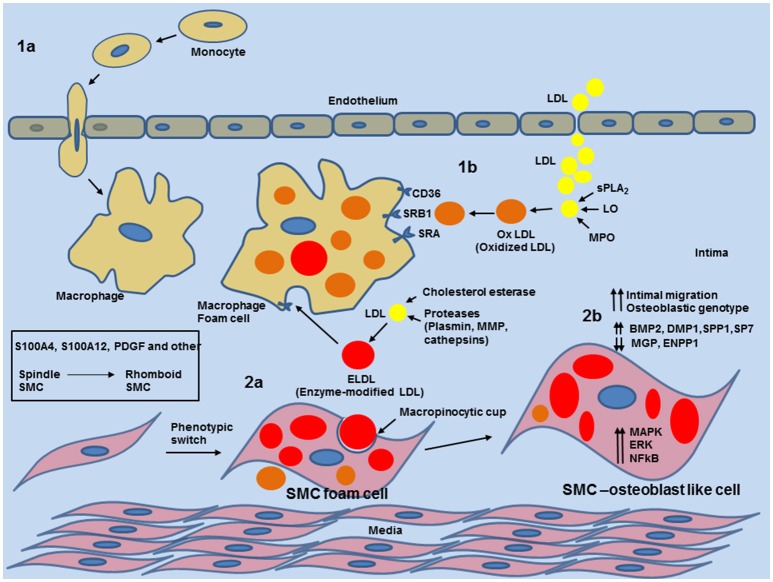
Schematic overview of phenotypic switch of contractile SMCs to osteoblast-like SMC by uptake of enzyme modified LDL in the medial layer and neo-intima in blood vessels. 1a: entry of circulating monocytes and transformation to macrophage foam cells provides enzymatic and inflammatory input for various modifications of native low density lipoprotein (LDL). 1b: Modification of LDL to oxidized LDL (oxLDL) by Phospholipase A2 (sPLA2), Lipoxygenase or Myeloperoxidase. oxLDL is readily taken by macrophages. 2a: Cholesterol esterase together with proteases (plasmin, metalloproteinases (MMPs) or cathepsin) generate non-oxidized enzymatic modified LDL (ELDL). ELDL is taken up by smooth muscle cells via macropinocytosis. 2b: uptake of ELDL activates cell signaling pathways (MAPK, ERK, NFkB) and downregulates mRNA for ectonucleotide pyrophosphatase/phosphodiesterase (ENPP-1), matrix gla protein (MGP) and upregulates mRNA for bone morphogenic protein 2 (BMP2), dentin matrix protein 1 (DMP1), osteopontin (SPP1), and osterix (SP7).

ELDL alone (10 μg/ml) or in combination with organic phosphate beta Glycerophosphate (10–30 mM) did not promote Alizarin Red staining of HCASMC when cultured up to 7 days, however, the addition of inorganic phosphate at low concentrations from 0.5 to 1.5 mM significantly induced mineralization of ELDL-treated cells as early as the second day of incubation (Figure 4). mRNA for genes known to promote calcification (BMP-2), DMP-1, ALPL, Runt-related transcription factor 2 (RUNX2), Osteopontin (OPN/SPP1), osterix/SP7 were increased in parallel with suppression of genes known to inhibit calcification (MGP, ENPP-1). Taken together, this demonstrated that ELDL uptake into SMCs resulted in a phenotypic to osteoblast-like cells as summarized in Figure 5.

Other models support the hypothesis that cholesterol metabolism in SMCs effects profoundly processes relevant to blood vessel remodeling, atherosclerosis and calcification. For example, low-density lipoprotein receptor-related protein 1 (LRP1) protects against cholesterol intracellular accumulation (76) and LDLR knockout mice with SMC specific deletion of LRP-1 showed increased foam cell formation, atherosclerosis and vascular remodeling with aneurysm formation (77). This was attenuated by pharmacological treatment with rosiglitazone, an agonist to the nuclear receptor PPARγ (78). But interestingly, despite advanced atherosclerosis in the LDLR knockout mouse with SMC- specific LRP1 deletion, there was no evidence of vascular calcification (79), suggesting that LRP1 in SMCs is required for vascular calcification. LRP1 deficient aorta had attenuated Wnt5a, and Wnt signaling pathways are critically important to drive osteochondrogenic differentiation (80, 81). Similar, LDLR knockout mice with PPARγ loss in SMC exhibit enhanced Wnt5a expression in the vascular wall and are susceptible to vascular calcification (79).

Recently, there has been growing interest in understanding the role of sortilin in vascular disease after the initial GWAS studies found the 1p13 locus harboring the SORT 1 gene encoding the protein sortilin to be associated with LDL, myocardial infarction, abdominal aortic aneurysms and CAC (82–85). Sortilin is a protein that regulates VLDL release from the liver and regulates lipid metabolism and inflammation. Mice with global deletion of sortilin have reduced serum levels of VLDL and are protected from atherosclerosis (86, 87). However, only recently it was shown by Dr. Aikawas group that the attenuated calcification in this mouse model is exclusively driven by loss of sortilin in SMCs by demonstrating that Sortilin is required for the assembly of tissue non-specific ALPL (TNAP) to extracellular vesicles released from SMCs (88). The release of extracellular vesicles from SMCs, a subpopulation known as matrix vesicles, containing calcium, phosphate, and ALPL are critically involved in medial calcification [reviewed in (18)]. Cholesterol is an integral component of cell membrane and matrix vesicles that nucleate calcium mineral, and thereby essential for matrix mineralization of SMCs. Geng et al. demonstrated that SMC deficient for LDL receptor and treated with mevastatin, a HMG-CoA inhibitor of cholesterol biosynthesis, failed to calcify *in vitro* (89). Collectively, these studies demonstrate elegantly how proteins regulating cholesterol metabolism have a dual role and control SMC-mediated calcification. This provides a potential explanation for the co-existence of atherosclerotic/intimal calcification with medical calcification and shared cellular mechanisms.

## Phosphate-induced calcification of SMCs

Hyperphosphatemia is associated with vascular calcification and this has been attributed to increases in spontaneous formation of calcium-phosphate crystals, induction of BMP-2 and other osteochodrogenic regulators, and downregulation of alpha smooth muscle actin (90).

Hydroxyapatite is the main calcium phosphate crystal found in bone and calcified tissue and is formed through intermediaries of phosphate and calcium. The high glycine content of extracellular matrix proteins collagen type 1 and elastic fibers favors the binding of calcium non-ionically, via charge neutralization to glycine. This enables phosphate to bind the calcium in a configuration that favors apatite formation. Pyrophosphate is a potent inhibitor of calcium crystallization and deposition. Pyrophosphate has been used industrially for this purpose and is the active ingredient in plaque-preventing toothpaste. Pyrophosphate acts by avidly binding to nascent hydroxyapatite crystals and inhibits hydroxyapatite crystal formation completely at micromolar concentrations.

Extracellular pyrophosphate is a potent physicochemical inhibitor of hydroxyapatite crystal formation *in vitro* (91) and *in vivo* (92–95). Pyrophosphate concentration is regulated by the ratio of synthesis to degradation, with the enzyme ectonucleotide pyrophophatase/phosphodiesterase 1 (ENPP1) generating pyrophosphate via hydrolysis of extracellular ATP, and degradation of pyrophosphate to inorganic phosphate is controlled by TNAP. Extracellular ATP and ADP is also hydrolyzed by the enzyme ectonucleoside triphosphate diphosphohydrolase 1 (ENTPD1, also known as CD39), releasing inorganic phosphate. By reducing the availability of extracellular ATP to generate pyrophosphate, ENTPD1 may be involved in vascular calcification, in addition to modulating inflammation (96). Lack of CD73, which acts downstream from CD39, by dissipating extracellular AMP to form adenosine, has also been implicated in human vascular calcification (97).

Pyrophosphate deficiency is the basis for vascular calcification in several genetic disorders (98, 99). ENPP1 encodes a cell surface protein that generates pyrophosphate by hydrolyzing extracellular ATP. A recessive loss of function mutation in *ENPP1* was shown to be causal for infantile arterial calcification, and infants born with this recessive disease develop arterial calcification in the first months of life (98–100). Moreover, the *ENPP1* gene had been previously linked to ectopic calcification through a mouse model known as the “tiptoe walking mouse” or *tww* (101).

Villa Bellosta et al. have shown that pyrophosphate synthesis increases 10 times more than pyrophosphate degradation during phosphate-induced calcification (90, 102). Hence, increased levels of ENPP1 mRNA or enzyme activity during calcification is a possible compensatory mechanism for cells to increase pyrophosphate concentration in an environment of phosphate overload and calcification, and should not be seen as a mediator of vascular calcification. This is supported by findings of widely calcified aortas in ENPP1 null mice (102). Since extracellular concentration of pyrophosphate is determined by the ratio of ENPP1/ENTPD1 and TNAP activity, with ENPP1 stimulating pyrophosphate synthesis and ENTPD1 and TNAP stimulating degradation of pyrophosphate, mRNA levels of all three enzymes should be monitored simultaneously during experimental calcification to gain insight into pyrophosphate regulation. TNAP expression is downregulated during early phases of calcification, prior to the accumulation of calcium, but is upregulated during later phases of calcification. These findings suggest that TNAP expression due to its biphasic response might need to be combined with other markers of calcification, like BMP2 mRNA, which was a reliable and sensitive marker of phosphate-induced calcification in cultured SMC and in calcified aorta, and parallels the earliest phases of vascular calcification (90). There is an substantial body of evidence linking TNAP upregulation and pyrophosphate deficiency to medial calcification associated with diabetes (103) and in patients undergoing dialysis (104). Moreover, TNAP is upregulated in the vasculature in several animal models of arterial calcification (92, 105–108). Transgenic mice with overexpression of TNAP in SMC show extensive vascular calcification, hypertension, cardiac hypertrophy and premature death, which was attenuated by TNAP inhibitor SBI-425 (109).

While our understanding of the biology of *ENPP1* and its role in ectopic and vascular calcification is likely to expand, studies implicate RAGE, the receptor for advanced glycation end products, as one pathway through which ENPP1 may act (50). *ENPP1* null mice in combination with loss of RAGE developed less arterial calcification and intra-arterial chondrogenic differentiation compared to *ENPP1* null mice with intact RAGE signaling, indicating an important role for oxidative stress and chronic inflammation as a modifier of phosphate-mediated calcification (50). Our finding that SMC stimulated with ELDL repress *ENPP1* gene expression is intriguing and suggests a possible key mechanism of ELDL-mediated calcification of SMCs.

There is substantial evidence for RAGE as a mediator of vascular disease (110), and importantly, mice lacking RAGE have attenuated atherosclerosis in apolipoprotein E deficient mice (51). S100A12 and the RAGE axis are emerging pathways linking inflammation to atherosclerosis and vascular calcification as shown by enhanced vascular calcification in S100A12 transgenic mice. Moreover, genetic ablation of S100A9 (111) or RAGE (51) resulted in reduced atherosclerosis in ApoE null mice. Pharmacological inhibition of S100A12 using Quinolone 3-carboxamide (ABR-215757), which binds S100A12 and prevents activation of RAGE *in vitro*, also reduces atherosclerosis and vascular calcification in ApoE null mice with transgenic S100A12 expression, suggesting that that S100A12 is a potential pharmacological target (31, 35). S100A12 may have a role as a biomarker of future cardiovascular disease (36), and may be able to be incorporated into primary prevention and risk factor modification (37). However, S100A12 has not been specifically targeted in prospective clinical trials.

**Figure 6:**
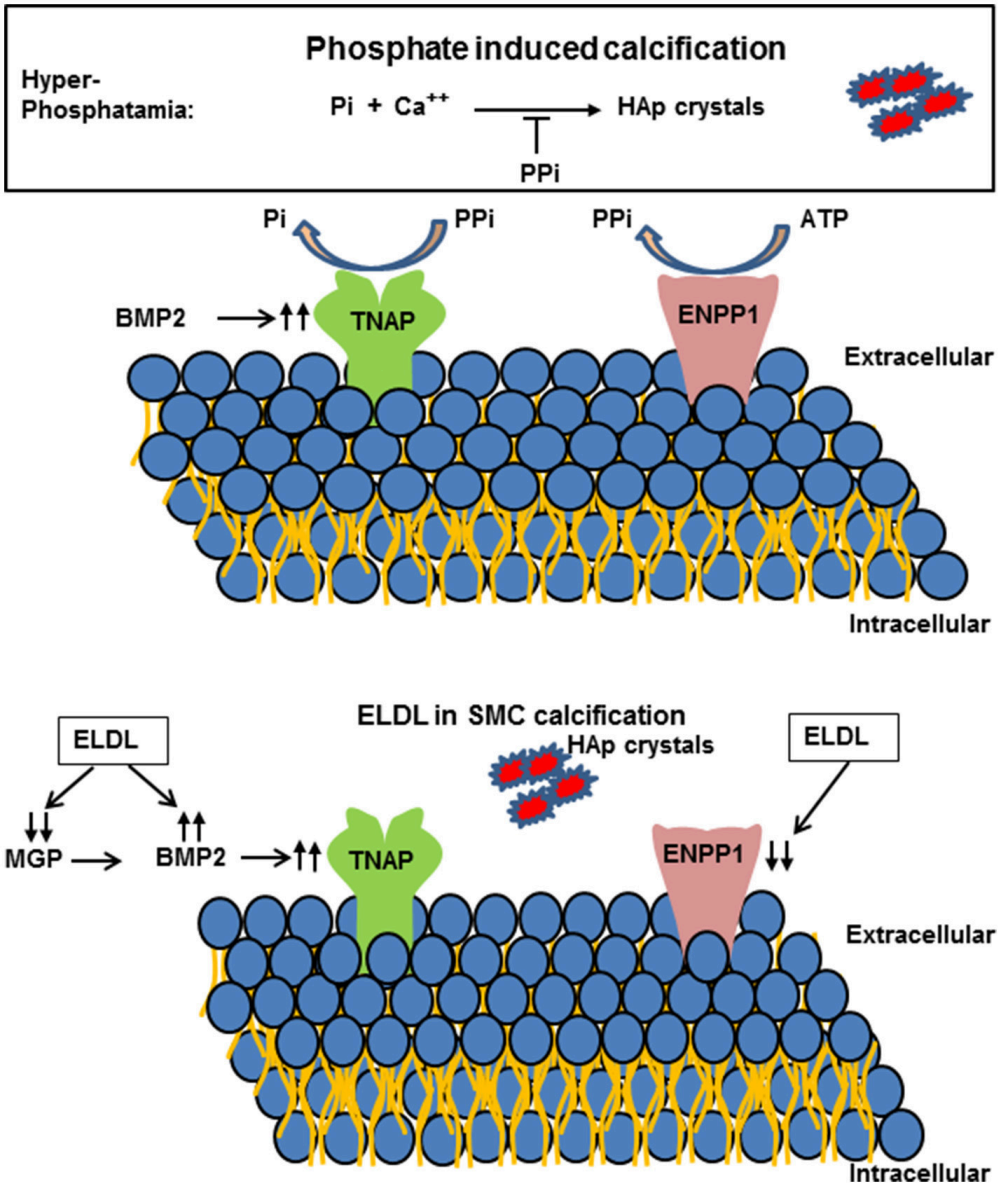
**(Upper)** Schematic representation of extracellular pyrophosphate metabolisms. Pyrophosphate (PPi) is an important inhibitor of Hydroxyapatide crystal formation. Ectonucleotide pyrophosphate phosphodiesterase 1 (ENPP1) hydrolyzes adenosine triphosphate (ATP) to PPi. PPi is degraded to phosphate (Pi) by tissue nonspecific alkaline phosphatase (TNAP). **(Lower)** Enzyme modified LDL (ELDL) reduces ENPP1 mRNA and other inhibitors (matrix gla protein, MGP) and increases bone morphogenetic protein 2 and thereby promotes calcification of cultured human coronary artery smooth muscle cells.

While there is no doubt of the importance of pyrophosphate as an physiologic inhibitor of vascular calcification (102), strategies to raise pyrophosphate levels by targeting its formation or metabolism (e.g., by augmenting ENPP1 or administering TNAP inhibitors) may hold promise as future therapies. Our data suggests that limiting of ELDL-mediated suppression of ENPP1 in smooth muscle could be a novel approach to the prevention of vascular calcification (Figure 6). ELDL and other oxidative and non-oxidative modifications of LDLs are most prevalent in chronic kidney disease and render lipoproteins more atherogenic (15, 102). A better understanding of steps leading to local production of ELDL in the vessel wall, its uptake by SMCs and subsequent phenotypic changes of SMCs may help to identify new targets for prevention of ectopic vascular calcification. Taken together, our data in HCASMCs in response to *in vitro*-generated ELDL supports the hypothesis that ELDL could be a critical mechanistic link between chronic inflammation and accelerated vascular calcification.

In summary, vascular calcification is complex and is driven by genetic as well as environmental exposures. While our understanding of this complex disorder has significantly progressed over the last two decades, much work remains in order to clarify mechanisms, particularly given that vascular calcification varies phenotypically, and may actually represent variations on a pathobiological theme. Prevention of calcification would be ideal, but reversing or limiting vascular calcification, once initiated, is another key strategy to mitigate the morbidity and mortality that occur as a result of vascular calcification. Given our current understanding of vascular calcification, strategies of prevention are more likely to translate into clinical practice than the more complex task of reversing vascular calcification. Therefore, any visualization of subclinical vascular calcification, either on a screening mammogram or on computed tomography should be seen as an opportunity to investigate risk factors and initiate preventive strategies. So perhaps the greatest power in screening mammography lies in the potential to prevent breast cancer and coronary artery disease from developing in the first place. Breast cancer and vascular calcification share several modifiable risk factors: diabetes mellitus, smoking, physical inactivity, poor diet, and obesity. If women were counseled each time they had a mammogram that controlling their risk factors could help them avoid two of the deadliest conditions for women, then the message of prevention could gain considerable strength (6).

## Author contributions

All authors listed have made a substantial, direct and intellectual contribution to the work, and approved it for publication.

### Conflict of interest statement

The authors declare that the research was conducted in the absence of any commercial or financial relationships that could be construed as a potential conflict of interest.
